# Diagnosis and management of ectopic ovarian pregnancy: a rare case report

**DOI:** 10.1016/j.ijscr.2021.106742

**Published:** 2021-12-29

**Authors:** Maryem Bouab, Ahmed Benjelloun Touimi, Mohamed Jalal, Amine Lamrissi, Karima Fichtali, Said Bouhya

**Affiliations:** Obstetrics and Gynecology Department, University Hospital Center Ibn Rochd, Casablanca, Morocco; Faculty of Medicine and Pharmacy, Hassan II University, Casablanca, Morocco

**Keywords:** Ovarian pregnancy, Diagnosis, Laparotomy, Case report

## Abstract

**Introduction:**

Ovarian pregnancy is a rare form of ectopic pregnancy. Its incidence is 0.5% to 1% of all ectopic gestations, or 1 in 7000 to 40,000 live births. The diagnosis is intricate and based on surgical and histopathological observations.

Traditional risk factors for ovarian ectopic pregnancy are similar to those for tubal pregnancy, but use of an IUD seems to be disproportionately associated.

**Case report:**

We report a rare case of spontaneous ectopic ovarian pregnancy of a 40-year-old woman, diagnosed with a ovarian pregnancy by ultrasound and treated by laparotomy in emergency obstetrical department of Ibn Rochd University Hospital of Casablanca.

**Clinical discussion:**

Ovarian ectopic pregnancies occur through fertilization of an egg retained in the peritoneal cavity leading to implantation on the surface of the ovary.

The increased incidence of ovarian ectopic pregnancies is associated with the increased use of artificial reproductive technologies (ART) and intrauterine contraceptive devices (IUCDs).

The diagnosis is difficult and a constant challenge for the gynecologist.

Its management remains surgical therapy despite the progress in medical treatment.

**Conclusion:**

Ovarian pregnancy is a rare entity that has some special features. Its diagnosis is difficult and relies on criteria based on intraoperative findings. Its management remains surgical therapy despite the progress in medical treatment.

## Highlights

• Ovarian ectopic pregnancy is a rare form of ectopic pregnancy and constitutes approximately 1% of all ectopic cases
• Definitive diagnosis & management of ovarian ectopic pregnancy is surgical laparoscopy/laparotomy and histopathology
• Early diagnosis and management is vital in order to prevent maternal morbidity and mortality

## 1 Introduction

Ovarian pregnancy is a rare form of ectopic pregnancy. Its incidence is 0.5% to 1% of all ectopic gestations, or 1 in 7000 to 40,000 live births. The diagnosis is intricate and based on surgical and histopathological observations [1].

Traditional risk factors for ovarian ectopic pregnancy are similar to those for tubal pregnancy, but use of an IUD seems to be disproportionately associated [2].

Ovarian ectopic pregnancies are associated to a high-risk of maternal morbidity and mortality.

We report the case of a patient with no particular pathological history primigravida, who has ovarian pregnancy successfully treated by laparotomy in emergency obstetrical department in Ibn Rochd University Hospital of Casablanca. This work has been reported with respect to the SCARE 2020 criteria [3].

## 2 A case presentation

**Fig. 1:**
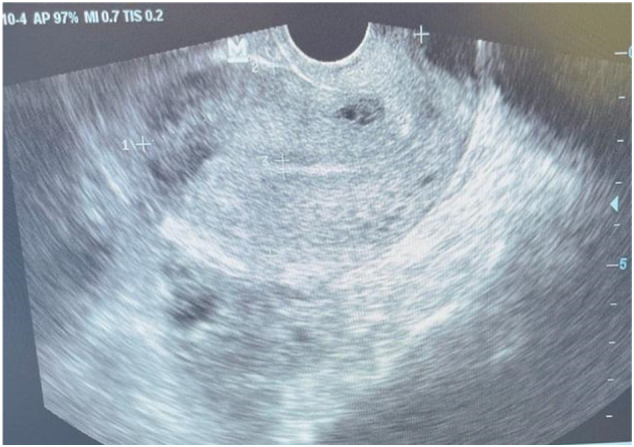
Ultrasound image of uterine vacuity.

**Fig. 2:**
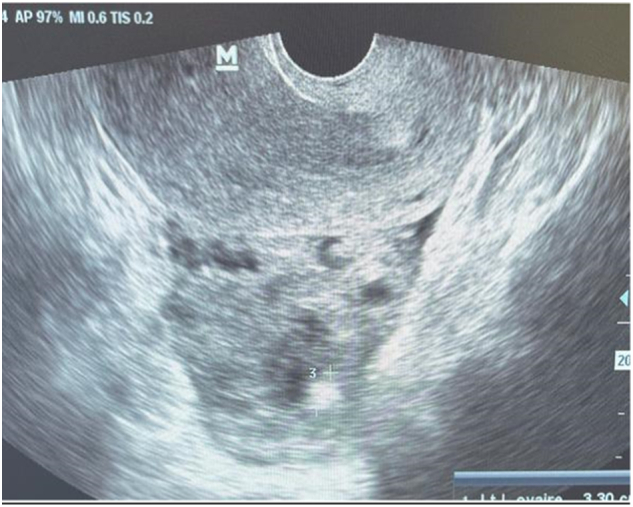
Ultrasound image of the gestational sac with embryo.

**Fig. 3:**
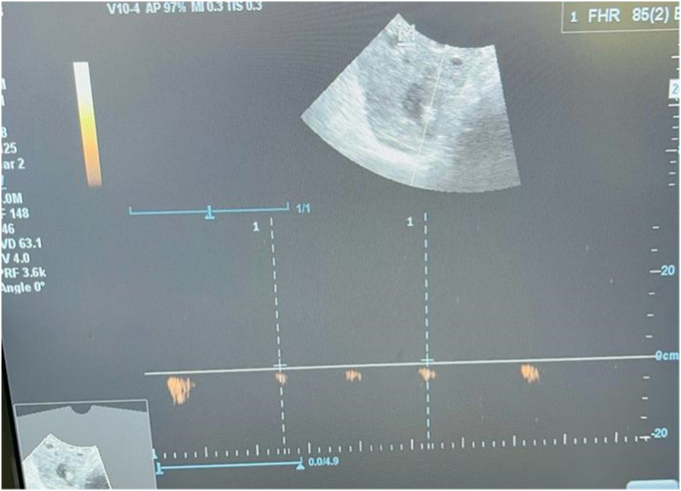
Ultrasound image of cardiac activity present of the embryo.

**Fig. 4:**
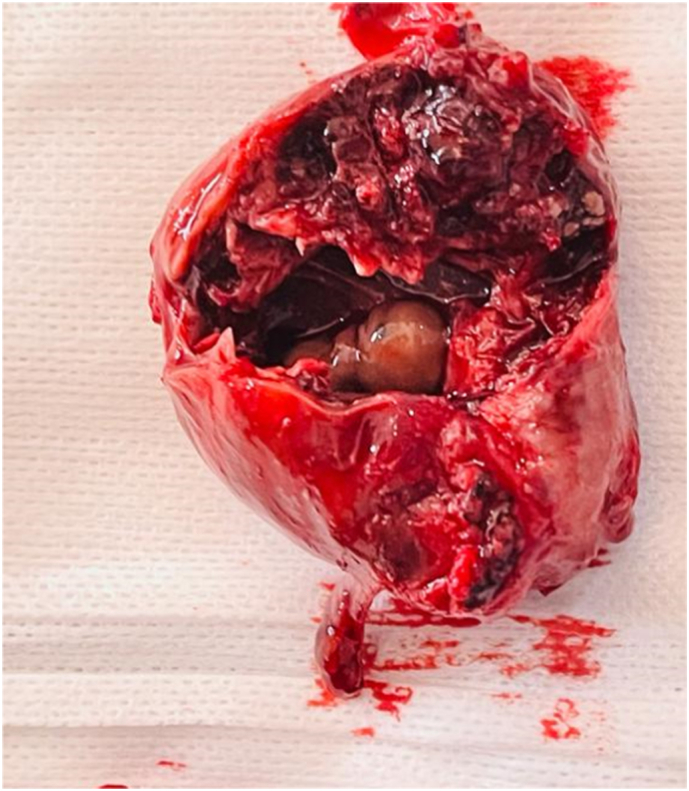
Visualization of the embryo after dissection of the ovary.

A 40-year-old patient with no particular pathological history, primigravida nulliparous, admitted for acute pelvic pain associated with blackish metrorrhagia with amenorrhea of 40 days, all evolving in a context of apyrexia and conservation of the general state. The examination on admission revealed a conscious patient, 15/15, with a blood pressure of 100/50 mmHg, tachycardia at 100 beats/min, the gynecological examination revealed an enlarged uterus with *endo*-uterine bleeding and a perceived left latero-uterine mass. The biological workup showed anemia at 10 g/dl, a BHCG level at 454 IU/ml. Pelvic ultrasound showed a left latero-uterine mass of 7 cm long axis with an embryo of 7 weeks of amenorrhea with cardiac activity, the uterus was empty with endometrial thickening of 25 mm, no pelvic effusion was found. (Fig. 1, Fig. 2, Fig. 3). The indication for laparotomy was given. On exploration, the presence of an unruptured left ovarian pregnancy was observed. Intraoperatively, we could not preserve the ovary, so a left adnexectomy was performed (Fig. 4). The post-operative course was simple. The patient was declared discharged after 4 days under oral contraception and iron supplementation.

## 3 Discussion

Although ovarian pregnancy is a rare entity, its incidence is increasing. The first reported case of ovarian pregnancy was described in the 17th century [4].

Historically, the reported incidence was 0.5% to 1% of all ectopic gestations, or 1 in 7000 to 40,000 live births [5].

Ovarian ectopic pregnancies occur through fertilization of an egg retained in the peritoneal cavity leading to implantation on the surface of the ovary [6].

Although the cause of these implantation anomalies remains uncertain, current hypotheses include reflux of the fertilized oocyte to the ovary, thickening of the tunica albuginea, and tubal dysfunction.

The increased incidence of ovarian ectopic pregnancies is associated with the increased use of artificial reproductive technologies (ART) and intrauterine contraceptive devices (IUCDs) [7].

The diagnosis is difficult and a constant challenge for the gynecologist.

Often it is misdiagnosed clinically and sonographically as a ruptured tubal ectopic pregnancy, corpus luteum cyst, hemorrhagic cyst and chocolate cyst of the ovary. It is even difficult to differentiate an ovarian pregnancy from a hemorrhagic ovarian cyst at the time of surgery [8].

Surgical criteria for the diagnosis of ovarian ectopic pregnancy have been described:

- fallopian tubes, including fimbria, must be intact and separate from the ovary,
- the pregnancy must occupy the normal position of the ovary,
- the ovary must be attached to the uterus through the utero-ovarian

ligament and

- there must be ovarian tissue attached to the pregnancy specimen [9] [10]

As demonstrated in the case discussed, the preoperative diagnosis of an ovarian ectopic pregnancy can be difficult because the symptoms are not specific and the ultrasound diagnosis is difficult [11].

Rupture in the first trimester is the usual rule in an ovarian ectopy, but the pregnancy may advance to full term [12].

Care must be taken not to mistake an ovarian ectopic pregnancy with other ovarian pathology.

However, no specific ultrasound criteria have been described, with ultrasound findings described in individual case reports [13].

Selection of treatment method — pharmacological treatment or surgery, preferably sparing, should be taken individually. The patients clinical condition, the results of additional tests, as well as her obstetric history and the desire for further procreation should be considered as well [14].

## 4 Conclusion

Ovarian pregnancy is a rare entity that has some special features. Its diagnosis is difficult and relies on criteria based on intraoperative findings. Its management remains surgical therapy despite the progress in medical treatment.

Now, with ultrasonographic advances, it can be diagnosed early, leading to conservative treatment and preservative surgery.

## Provenance and peer review

Not commissioned, externally peer-reviewed.

## Consent

Written informed consent for publication of their clinical details and/or clinical images was obtained from the patient.

## Ethical approval

I declare on my honor that the ethical approval has been exempted by my establishment.

## Sources of funding

None.

## Guarantor

Dr. Bouab Maryem

## Registration of research studies

None.

## CRediT authorship contribution statement

Dr. Bouab Maryem: Corresponding author, writing the paper.

## Declaration of competing interest

The authors declare having no conflicts of interest for this article.
